# Genomic Locus Modulating IOP in the BXD RI Mouse Strains

**DOI:** 10.1534/g3.118.200190

**Published:** 2018-03-01

**Authors:** Rebecca King, Ying Li, Jiaxing Wang, Felix L. Struebing, Eldon E. Geisert

**Affiliations:** * Department of Ophthalmology, Emory University, Atlanta, Georgia 30322; † Department of Ophthalmology, Tianjin Medical University General Hospital, Tianjin, China

**Keywords:** BXD, cadherin, eye, glaucoma, intraocular pressure, IOP, mouse, quantitative trait mapping

## Abstract

Intraocular pressure (IOP) is the primary risk factor for developing glaucoma, yet little is known about the contribution of genomic background to IOP regulation. The present study leverages an array of systems genetics tools to study genomic factors modulating normal IOP in the mouse. The BXD recombinant inbred (RI) strain set was used to identify genomic loci modulating IOP. We measured the IOP in a total of 506 eyes from 38 different strains. Strain averages were subjected to conventional quantitative trait analysis by means of composite interval mapping. Candidate genes were defined, and immunohistochemistry and quantitative PCR (qPCR) were used for validation. Of the 38 BXD strains examined the mean IOP ranged from a low of 13.2mmHg to a high of 17.1mmHg. The means for each strain were used to calculate a genome wide interval map. One significant quantitative trait locus (QTL) was found on Chr.8 (96 to 103 Mb). Within this 7 Mb region only 4 annotated genes were found: *Gm15679*, *Cdh8*, *Cdh11* and *Gm8730*. Only two genes (*Cdh8* and *Cdh11*) were candidates for modulating IOP based on the presence of non-synonymous SNPs. Further examination using SIFT (Sorting Intolerant From Tolerant) analysis revealed that the SNPs in *Cdh8* (Cadherin 8) were predicted to not change protein function; while the SNPs in *Cdh11* (Cadherin 11) would not be tolerated, affecting protein function. Furthermore, immunohistochemistry demonstrated that CDH11 is expressed in the trabecular meshwork of the mouse. We have examined the genomic regulation of IOP in the BXD RI strain set and found one significant QTL on Chr. 8. Within this QTL, there is one good candidate gene, *Cdh11*.

Glaucoma is a diverse set of diseases with heterogeneous phenotypic presentations associated with different risk factors. Untreated, glaucoma leads to permanent damage of axons in the optic nerve and visual field loss. Millions of people worldwide are affected (European Glaucoma Prevention Study *et al.* 2007; Leske *et al.* 2007) and it is the second leading cause of blindness in the United States (Medeiros *et al.* 2003). Adult-onset glaucoma is a complex collection of diseases with multiple risk factors and genes with differing magnitudes of effects on the eventual loss of RGCs. The severity of the disease appears to be dependent on the interaction of multiple genes, age, and environmental factors (Herndon *et al.* 2004). There are also a number of phenotypic risk factors for POAG including: age, ethnicity, central corneal thickness and axial length (Gordon *et al.* 2002). The primary risk factor is an elevated intraocular pressure (IOP) (Klein *et al.* 2004). There are known genetic mutations that affect IOP that result in inherited glaucoma (Stone *et al.* 1997; Wiggs 2007). The prime example is MYOC, a protein secreted by the trabecular meshwork and mutations in this protein cause ER stress which results in a decrease in the function of the trabecular meshwork and an elevation in IOP (Joe *et al.* 2003; Kasetti *et al.* 2016). We know a considerable amount about the regulation of IOP from the production of aqueous humor to the outflow pathways. IOP is a complex trait affected by different tissues in the eye each of which is regulated by multiple genomic loci. Interestingly, there are very few studies (Springelkamp *et al.* 2014; Nag *et al.* 2014; Chen *et al.* 2014; Choquet *et al.* 2017; Ozel *et al.* 2014; Springelkamp *et al.* 2017) that have identified genomic loci in humans modulating normal IOP.

In the present study, we are using the BXD recombinant inbred (RI) strain set that is particularly suited for the study of genetics and the effects on the severity of glaucoma. This genetic reference panel presently consists of over 202 strains (Peirce *et al.* 2004), and we are now in the unique position of being able to study the eyes in mice with shuffled genomes from the two parental strains, C57BL/6J and the DBA/2J. There are over 7,000 break points in our current set of BXD strains. For this study, our group has measured IOP of 506 eyes from 38 strains to identify genomic loci modulating IOP. A systems genetics approach to glaucoma is a relatively new branch of quantitative genetics that has the goal of understanding networks of interactions across multiple levels that link DNA variation to phenotype (Mozhui *et al.* 2008). Systems genetics involves an analysis of sets of causal interactions among classic traits such as IOP, networks of gene variants, and developmental, environmental, and epigenetic factors. The main challenge is the need for comparatively large sample sizes and the use of more advanced statistical and computational methods and models. We finally have a sufficiently large number of strains to use this approach (Geisert *et al.* 2009; Freeman *et al.* 2011). Our goal is now to combine data across several levels from DNA to ocular phenotype and analyze them with newly developed computational methods to understand pre-disease susceptibility to glaucoma along with the genetic networks modulating the response of the eye to elevated IOP.

## Methods

### Mice

This study measured the IOP in the 36 BXD strains of mice along with the parental strains, the C57BL/6J mouse strain and the DBA/2J mouse strain. None of the BXD strains included in this study carried both mutations (*Tyrp1* and *Gpnmb*) known to cause the severe glaucoma phenotype observed in the DBA2/J strain. All of the mice in this study were between 60 and 120 days of age, a time before there is any significant elevation in IOP due pigment dispersion (Anderson *et al.* 2002). The data presented in this paper is based on measurements from 506 eyes with roughly equal numbers of male and female mice. All breeding stock was ordered from Jackson Laboratories (Bar Harbor, ME) and maintained at Emory. Mice were housed in the animal facility at Emory University, maintained on a 12 hr light/dark cycle (lights on at 0700), and provided with food and water *ad libitum*. IOP measurements were made between 0900 and 1100. Both eyes were measured and the data from each eye was entered into the database. An induction–impact tonometer (Tonolab Colonial Medical Supply) was used to measure the IOP according to manufacturer’s instructions and as previously described (Saleh *et al.* 2007; Nagaraju *et al.* 2009). Mice were anesthetized with Avertin (334 mg/kg) or ketamine/xylazine (100,15mg/kg). Three consecutive IOP readings for each eye were averaged. IOP readings obtained with Tonolab have been shown to be accurate and reproducible in various mouse strains, including DBA/2J (Wang *et al.*, 2005). All measurements were taken approximately 10 min after the induction of anesthesia. These IOP measurements were made on mice prior to two different experimental procedures, blast injury to the eye (Struebing *et al.* 2018a) or elevation of IOP by injection of magnetic beads into the anterior chamber (Struebing *et al.* 2018b). All of the measurements were made on normal eyes. Previous studies have found significant effects of anesthesia on IOP (Ding *et al.* 2011; Qiu *et al.* 2014). When we compared the IOP of all mice anesthetized with Avertin to those anesthetized with ketamine/xylazine over the entire dataset there was no significant difference between the two groups. We specifically examined the C57BL/6J mouse strain, with 11 mice anesthetized with Avertin (mean IOP 10.2, SD 0.15) and 27 mice anesthetized with ketamine/xylazine (mean IOP 11.2, SD 2.9). Although there was a trend for the ketamine/xylazine anesthetized mice to have higher IOP, there was no statistically significant difference between the two groups using the Mann-Whitney *U*-test.

### Interval Mapping of IOP Phenotype

The IOP data will be subjected to conventional QTL analysis using simple and composite interval mapping along with epistatic interactions. The means for each BXD strain were calculated and these means were used to generate genome-wide interval maps. All of these data are available on GeneNetwork.org. Genotype was regressed against each trait using the Haley-Knott equations implemented in the WebQTL module of GeneNetwork (Chesler *et al.* 2005; Rosen *et al.* 2003; Carlborg *et al.* 2005). Empirical significance thresholds of linkage are determined by permutations (Churchill and Doerge 1994). We correlated phenotypes with gene expression data for whole eye and retina (Geisert *et al.* 2009; King *et al.* 2015; Templeton *et al.* 2013).

### Immunohistochemistry

For immunohistochemical experiments mice were deeply anesthetized with a mixture of 15 mg/kg of xylazine (AnaSed) and 100 mg/kg of ketamine (Ketaset) and perfused through the heart with saline followed by 4% paraformaldehyde in phosphate buffer (pH 7.3). The eyes were embedded in paraffin as described by Sun *et al.* (Sun *et al.* 2015). The eyes were dehydrated in a series of ethanol and xylenes changes for 20 min each (50% ETOH, 70% ETOH, 90% ETOH, 95% ETOH, two changes of 100% ETOH, 50% ETOH with 50% xylenes, two changes of 100% xylene, two changes of paraffin). The eyes were then embedded in paraffin blocks. The eyes were sectioned with a rotary microtome at 10µm and mounted on glass slides. Paraffin was removed from the sections and the sections were rehydrated. The sections were rinsed in PBS, and then placed in blocking buffer containing 2% donkey serum, 0.05% DMSO and 0.05% Triton X-100 for 30 min. The sections were rinsed in PBS, and then placed in blocking buffer containing 2% donkey serum, 0.05% DMSO and 0.05% Triton X-100 for 30 min. The sections were incubated in primary antibodies (1:500) against Cadherin 11 (Thermofisher, Cat. #71-7600, Waltham, MA) overnight at 4°. After rinsing, the sections were incubated with secondary antibody conjugated to AlexaFluor-488 (donkey anti-rabbit, Jackson Immunoresearch Cat #711-545-152, Westgrove, PA), (1:1000), for 2 hr at room temperature. The sections were then rinsed 3 times in PBS for 15 min each. Then they were counterstained with TO-PRO-3 iodide purchased from Invitrogen (T3605, Invitrogen, Eugene OR). The slides were flooded with Fluoromount-G (SouthernBiotech Cat #. 0100-01, Birmingham, AL), and covered with a coverslip. All images were photographed using a Nikon Eclipse TE2000-E (Melville, NY) confocal microscope and images were acquired by Nikon’s EZ-C1 Software (Bronze Version, 3.91).

### PCR Validation

Reverse transcription-quantitative polymerase chain reaction (RT-qPCR) were used to validate the mRNA expression level of *Cdh11* and *Cdh8* and *Myoc* in whole eyes of C57BL/6J mice. Primers were designed for *Cdh11*, *Cdh8* and *Myoc* using Primer BLAST-NCBI so that predicted PCR products were approximately 150bp long. Sequences of the PCR primers are listed in Table 1. PCR reactions were carried out in triplicate in 10μl reactions containing 5μl of 2x QuantiTect SYBR Green PCR Master Mix (Qiagen, Cat #204141 Hilden, Germany), 0.5 μl of forward primer (0.5μM), 0.5 μl of reverse primer (0.5 μM), 2μl of template cDNA(10ng) and 2μl of RNA free H_2_O. PCR of mouse genes was performed using a program beginning at 95° for 15 min, followed by 40 cycles of reaction with denaturation at 94° for 15 sec, annealing at 59° for 30 sec and extension at 72° for 30 sec of each cycle. The cycle threshold (Ct) values were normalized to a mouse housekeeping gene peptidylprolyl isomerase A (*Ppia*) to generate Delta Ct values (ΔCt) for each gene. Fold changes (FC) were calculated from the differences of each gene when compared with *Cdh11(*ΔΔCt) using the formula FC= 2^(-ΔΔCT). Data were represented as mean ± SE of mean. Four biological independent samples were used for statistical analysis using Mann-Whitney-*U*-test (Figure 4).

**Table 1:** Primers designed for Cdh11 and Cdh8 and Myoc

**Cdh11**	Forward 5′ GAAACCAAAGTCCCAGTGGCC 3′
	Reverse 5′ TGGTCCATTGGCTGTGTCGT 3′
**Cdh8**	Forward 5′ AGCCTCCGGTCTTCTCTTCAC 3′
	Reverse 5′ CAGTGTGGCGGTCAATGGAAA 3′
**Myoc**	Forward 5′ GCTGGCTACCACGGACACTT 3′
	Reverse 5′ CGCTCAAGTTCCAGGTTCGC 3′
**Ppia**	Mm_Ppia_1_SG QuantiTect Primer Assay

### Data Availability

All of the IOP data used in this study is presented in Table S1. The means from each strain and the standard deviations are provided in Table S2. The mapping file (Record ID 19520) for the data present in this paper can be found on GeneNetwork.org.

## Results

**Figure 1:**
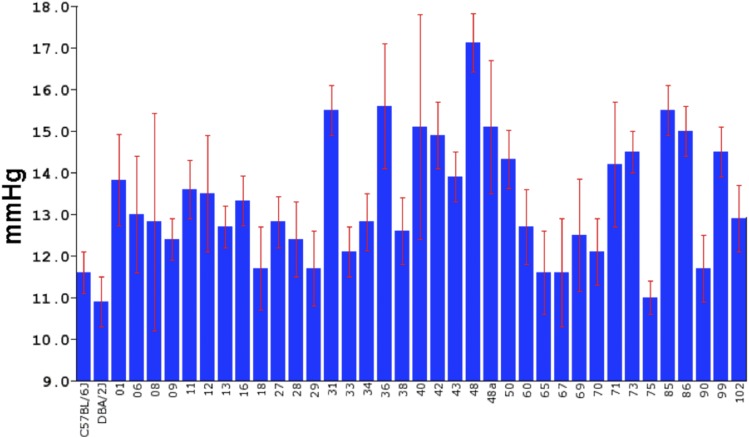
The distribution of IOP across the BXD strains is illustrated in a bar chart with means and Standard Error of the Mean In the 38 strains of mice the IOP ranged from a low of 10.9 mmHg to a high of 17.1 mmHg.

The overall goal of the present investigation was to determine if specific genomic loci modulate IOP in the BXD RI strains. IOP was measured in 506 eyes from 38 BXD RI strains and the two parental strains C57BL/6J mouse and DBA2/J mouse. To create a mapping file the strain averages and standard errors were calculated (Figure 1). The mean IOP measured across the 38 strains was 13.2 mmHg and the standard error of the mean was 1.5 mmHg. The strain with the lowest IOP was DBA2/J, with an average IOP of 10.9 mmHg. The strain with the highest IOP was BXD48 with an average IOP of 17.1 mmHg. The IOP of the parental strains was 11.6 mmHg for the C57BL/6J and 10.9 mmHg for the DBA2/J. This is a substantial amount of genetic transgression across the BXD RI strain set. This type of phenotypic variability is a clear indication that IOP is in fact a complex trait. These data can also be used to calculate the heritability of IOP. Figure 1 reveals a considerable variability in the IOP from strain to strain and the standard error for each strain is rather small. This type of data suggests that the genetic variability has a greater effect than the environmental variability. These data can be used to calculate the heritability H^2^ of IOP. H^2^ is the genetic variance (Vg) of the trait divided by the sum of genetic variance plus the environmental variance (Vg +Ve). The genetic variance can be estimated by taking the standard deviation of the mean of IOP for each strain (Vg = 1.7 mmHg). The environmental variance can be estimated by taking the mean of the standard deviation across the strain (Ve = 3.2 mmHg). Using the formula for heritability, H^2^ = Vg/(Vg + Ve), the calculation of 1.7 mmHg /(1.7 mmHg + 3.2 mmHg) reveals that H^2^ = 0.35. Thus, IOP is a heritability trait in the BXD RI strain set.

### Genome Wide Mapping

**Figure 2:**
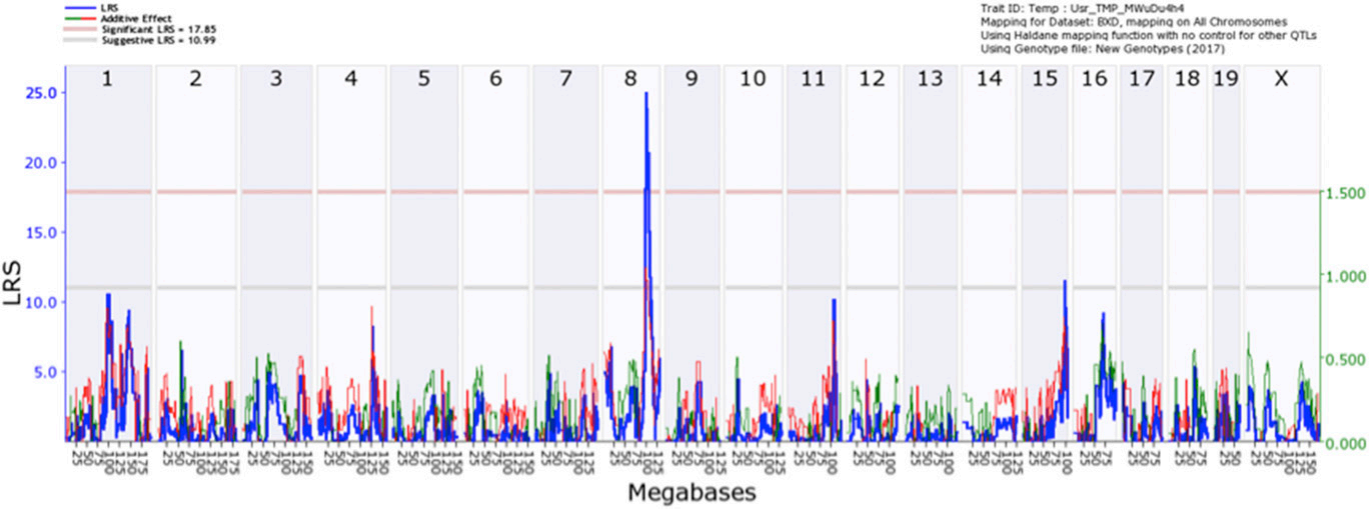
A genome-wide interval map of IOP. The interval map plots the likelihood ratio statistic (LRS) across the genome from chromosome 1 to chromosome X. The light gray line is the suggestive level and the light red line is genome-wide significance (*P* = 0.05). When the IOP measures were mapped to the mouse genome there was a significant association between IOP and a locus on Chromosome 8.

**Figure 3:**
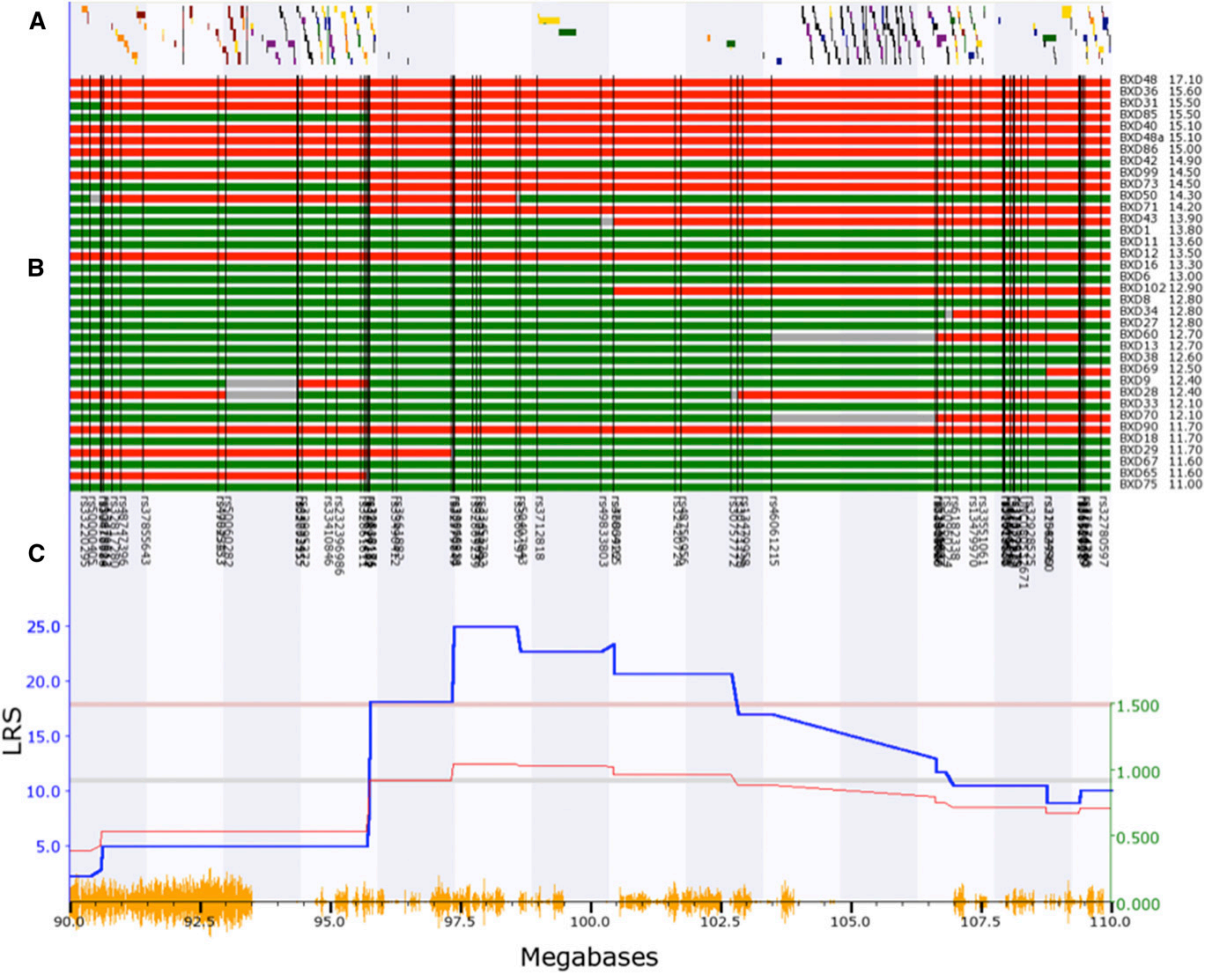
The interval map for Chr. 8: 90 to 110 Mb is illustrated. A is the gene tract, that identifies the locations of known genes across the genome. B is a haplotype map for the different BXD RI strains listed to the right and ranked from the highest IOP to the lowest IOP. The location of genomic markers is indicated by black vertical lines. C is an expanded version of the interval map for IOP. Finally, the bottom trace (yellow) identified the location of SNPs between the C57BL/6 mouse and the DBA2/J mouse. The genomic location is indicated along this lower trace. Notice that the peak of the QTL in C sits in a region of the genome that contains very few known genes (A).

Taking the average IOP from 38 strains of mice we performed an unbiased genome-wide scan to identify genomic loci (QTL) that modulate IOP. The genome-wide interval map (Figure 2) identifies one significant peak on Chr. 8. Examining an expanded view of Chr.8, 90 to 110 Mb (Figure 3), the peak of the IOP QTL reaches significance from the genomic marker rs3661882 (96.2 Mb) to rs13479958 (103 Mb). BXD strains with higher IOPs (Figure 3B) tend to have the C57BL/6J allele (red) and strains with lower IOPs tend to have the DBA2/J allele (green). When the distribution of genes within this region is examined (gene track Figure 3A) the significant portion of the QTL peak covers a region of the genome that is a gene desert. Within this region there are only 4 annotated genes: *Gm15679* (predicted gene 15679, Chr.8: 99.01- 99.03Mb), *Cdh8* (cadherin 8, Chr.8: 99.03-99.42Mb), *Cdh11* (cadherin11, Chr.8:102.63-102.79Mb) and *Gm8730* (predicted gene 8730, Chr.8: 102.86- 102.87Mb). Using the tools available on GeneNetwork (genenetwork.org) we are able to identify potential candidates for modulating IOP in the BXD RI strains. The candidate genes can either be genomic elements with *cis*-QTL or they can be genes with nonsynonymous SNPs changing protein sequence and affecting protein function. Within this region there are only two putative candidate genes. None of the candidate genes had valid *cis*-QTL in either the eye dataset (Geisert *et al.* 2009) or the DoD normal retinal dataset on GeneNetwork (King *et al.* 2015). Two of the genes in this region had non-synonymous SNPs: *Cdh11* and *Cdh8*.

For the initial evaluation of the two candidate genes we examined their expression level in a microarray dataset hosted on GeneNetwork: the eye database (Eye M430v2 (Sep08) RMA). In the eye dataset, the highest level of expression for a valid *Cdh11* probe set (1450757_at) is 10.8 Log_2_. This dataset is on a Log_2_ scale with the mean mRNA expression set to 8. Thus, Cdh11 is expressed at approximately eightfold above the mean. For *Cdh8* the highest expression of a valid probe set (1422052_at) is 7.3 Log_2_ or approximately twofold below the mean. These data demonstrate that *Cdh11* is expressed at a level over eightfold higher in the whole eye relative to the expression of *Cdh8*.

**Figure 4:**
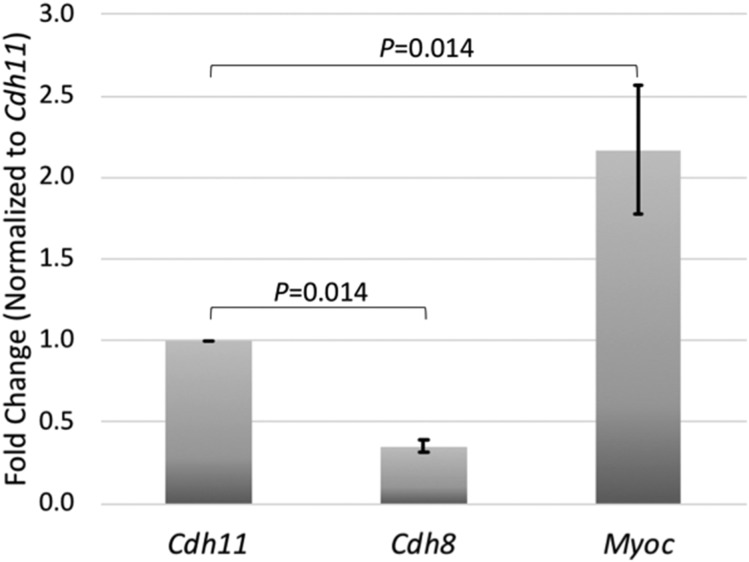
Expression of *Cdh11*, *Cdh8* and *Myoc* in whole eye. The mRNA expression levels of *Cdh11*, *Cdh8* and *Myoc* in mice whole eye are shown as fold changes normalized to *Cdh11*. Significant changes in gene expression were observed using a Mann-Whitney *U*-test.

To confirm the expression levels of *Cdh11* and *Cdh8* in the eye, we examined the levels of mRNA in the whole eye by RT-qPCR (Figure 4). In 4 biological replicate samples, we examined the levels of *Cdh11*, *Cdh8* and *Myoc* (a marker of trabecular meshwork cells, (Takahashi *et al.* 1998)). Our PCR analysis confirmed the general findings of the microarray data sets. In the 4 biological samples of whole eye, *Cdh11* was more highly expressed than *Cdh8*. The average of the 4 samples demonstrated a more than twofold higher expression of *Cdh11* (ΔCt = 5.75 ± 0.27) than *Cdh8* (ΔCt = 7.36 ± 0.33). *Myoc* (ΔCt = 5.27 ± 0.38) was expressed at a higher level than both *Cdh8* and *Cdh11*. All of these data taken together indicate that *Cdh11* is the prime candidate for an upstream modulator of IOP in the mouse. Using the RNA-seq data presented in the study by Carnes *et al.* (Carnes *et al.* 2018), we examined the expression level of *CDH11* and *CDH8* in the human trabecular meshwork. *CDH11* is highly expressed in adult human trabecular meshwork; while *CDH8* is expressed at very low levels if at all. The levels of *CDH11* are more than 55 times higher than that for *CDH8*(FPKM_CDH11_ = 88.5 *vs.* FPKM_CDH8_ = 1.6, FPKM: Fragments Per Kilobase Million). This remains true for fetal human trabecular meshwork (FPKM_CDH11_ = 270.1 *vs.* FPKM_CDH8_ = 4.6).

To determine if any of the non-synonymous SNPs in either *Cdh8* or *Cdh11* could affect protein function, we ran a SIFT (Sorting Intolerant From Tolerant) analysis (Kumar *et al.* 2009). The SNP in *Cdh8* (rs37017336 cysteine to arginine) had a SIFT value of 0.19 indicating that this mutation in the protein would be tolerated, not affecting protein function. The two SNPs in *Cdh11* (rs30742273 valine to methionine, and rs33464298 glutamic acid to aspartic acid) both had SIFT values of 0.03, predicting that each of these SNPs would not be tolerated and would be deleterious to protein function. All of these data point to *Cdh11* as the one good candidate gene.

### Distribution of Cadherin 11 in the Eye

**Figure 5:**
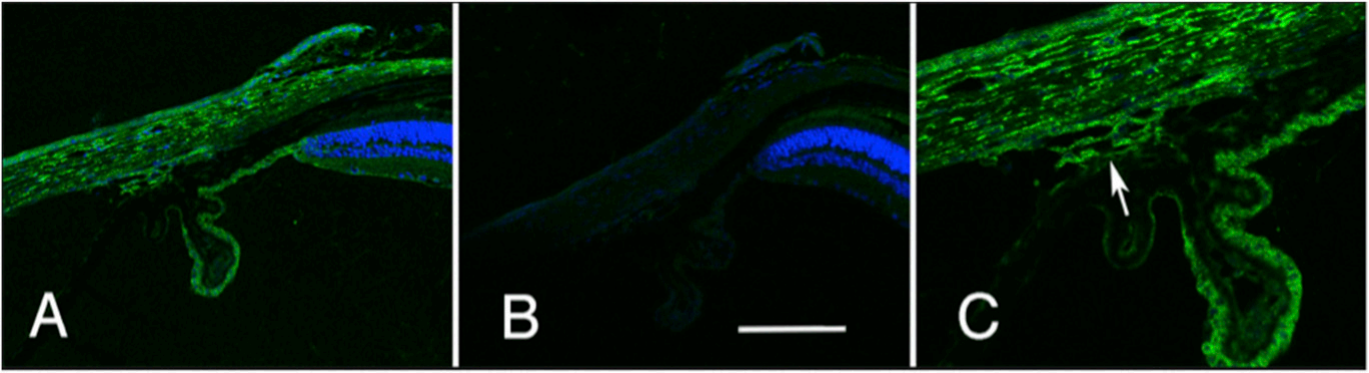
The distribution of cadherin 11 in the limbal area of the eye is illustrated. The section in A was stained with an antibody specific to cadherin 11 (green) and for DNA (blue). This staining is specific to the primary antibody for it is not observed in a section stained with the secondary antibody alone (B). The staining pattern of the trabecular meshwork is shown at higher magnification in C (arrow). A and B are taken at the same magnification and the scale bar in panel B represents 25 µm.

To determine if Cadherin 11 is found in structures associated with the control of IOP, we stained sections of the eye for Cadherin 11. In these sections, there was antibody-specific staining of several structures (Figure 5A) as demonstrated by the lack of staining in control sections stained with secondary antibody only (Figure 5B). There is extensive labeling of all layers of the cornea. The epithelium of the ciliary body is also heavily labeled as well as labeling of pars plana. There is also light labeling of the retina. At higher magnification (Figure 5C), clear labeling of the trabecular meshwork (arrow) is observed. These data reveal that the expression pattern of Cadherin 11 in the mouse eye and its specific expression in the cells of the trabecular meshwork is appropriate for a protein involved in regulation of IOP.

## Discussion

The normal regulation of intraocular pressure is a balance between production in the ciliary body and outflow (Brubaker 1991; Goel *et al.* 2010). In the human, IOP can range from a relatively low pressure to an extremely high one that occurs in acute angle closure glaucoma. It is generally accepted that the “normal” range for IOP in humans is from 12mmHg to 22mmHg (Hollows and Graham 1966; Renard *et al.* 2010). In addition, monitoring throughout the day reveals IOP is pulsatory and has a diurnal variability (Aptel *et al.* 2016). These findings tell an interesting story about the regulation of pressure in the eye; however, the primary driving force behind the intense investigation of IOP in humans is the fact that it is the primary risk factor for developing glaucoma (Sommer *et al.* 1991). Furthermore, all of the current treatments for glaucoma center around lowering IOP either by pharmacological approaches or surgery (Cohen and Pasquale 2014; Gedde *et al.* 2013).

The known association between elevated IOP and glaucoma has driven most of the study of IOP in human populations (Gordon *et al.* 2002; Ojha *et al.* 2013; Ozel *et al.* 2014). Most of these studies involve the study of glaucoma, but a few have a primary focused on the regulation of IOP. These studies have found that IOP is a heritable trait with estimates of heritability ranging from 0.39 to 0.64 (Klein *et al.* 2004; Chang *et al.* 2005; Carbonaro *et al.* 2009; van Koolwijk *et al.* 2007). In the present study, we found that the heritability of IOP in the BXD RI strains was 0.35. Thus, the mouse strains demonstrated a heritability near the lower end of the human populations. The interest has prompted studies to identify genes regulating IOP. In a genome-wide association study of IOP involving 11,972 subjects, significant associations were observed with SNPs in two genes, *GAS7* and *TMCO1* (van Koolwijk *et al.* 2012). Both of these genes are expressed at high levels in the ciliary body and trabecular meshwork (Liton *et al.* 2006) and both of the genes interact with known glaucoma risk genes (van Koolwijk *et al.* 2012). TMCO1 is also known to be associated with severe glaucoma risk (Burdon *et al.* 2011).

In an effort to understand the regulation of IOP and its effects on the retina, many research groups have used inbred mouse strains (Savinova *et al.* 2001; Struebing and Geisert 2015; Sappington *et al.* 2010; Cone *et al.* 2010; Samsel *et al.* 2011; Chintalapudi *et al.* 2017). IOP varies widely across different strains of mice (Savinova *et al.* 2001; Wang *et al.* 2005), ranging from a low of 11mmHg in the BALB/c mouse strain to a high of 19mmHg in the CBA/Ca mouse strain. In the present study, the average measured IOP across the 34 strains was 13.2mmHg. The lowest measured IOP was 10.9mmHg in the DBA/2J strain and the highest was 17.1mmHg in the BXD48 strain. All of these studies are complicated by the difficulties of measuring IOP in the mouse (Wang *et al.* 2005; Ding *et al.* 2011). The present study examines IOP in the anesthetized mouse and there is substantial evidence that anesthesia can affect IOP (Ding *et al.* 2011; Qiu *et al.* 2014). That being said the present study relies on the variability across the BXD RI strains. All of the current data suggests that anesthesia affects the absolute IOP value; however, the relative differences in IOP between BXD strains is sufficient to map a QTL. Thus, we were able to use these measures to map a single significant QTL on Chr. 8 that modulates IOP in the mouse. The peak of the QTL was in a gene desert and within this region there were only two potential candidate genes that could be modulating IOP in the BXD strain set. Based on expression of mRNA in the eye microarray dataset, the findings of real time PCR and functional analysis of SNPs, *Cdh11* appears to be the best candidate. *Cdh11* is expressed approximately eightfold higher in the eye than is *Cdh8*. A recent study of human trabecular meshwork using RNA Seq found that *CDH11* is expressed at relatively high levels and at least 55-fold higher that the expression of *CDH8* (Carnes *et al.* 2018). Furthermore, *CDH11* is highly expressed in cultured human trabecular meshwork cells (Paylakhi *et al.* 2012). We found that Cadherin 11 is expressed in the trabecular meshwork of the mouse using indirect immunohistochemistry. All of these data suggest that the expression of Cadherin 11 in the trabecular meshwork modulates IOP across the BXD RI strain set.

How is it possible that a cadherin can modulate IOP in the mouse eye? IOP is regulated by fluid resistance at the trabecular meshwork and Schlemm’s canal (Ethier *et al.* 1986; Brubaker 1975). The stiffness of these structures is determined by the extracellular matrix within the trabecular meshwork and Schlemm’s canal (Zhou *et al.* 2012). The contractile nature of the cells within the inner wall is considered to play an important role in regulating outflow resistance (Bradley *et al.* 1998; Johnson 2006; Vranka *et al.* 2015). The dysregulation or poor organization of extracellular matrix may increase the fluid resistance, leading to an elevation of the IOP. *Cdh11* was recently revealed to be a novel regulator of extracellular matrix synthesis and tissue mechanics (Row *et al.* 2016), and it is also found to be highly expressed in cultured human trabecular meshwork cells (Paylakhi *et al.* 2012). It is possible that the IOP can be regulated by *Cdh11* and related pathways by altering the extracellular matrix structure of the trabecular meshwork. Future studies about the role of *Cdh11* in the trabecular meshwork may give insights into the mechanism of IOP modulation.

## Supplementary Material

Supplemental Material is available online at www.g3journal.org/lookup/suppl/doi:10.1534/g3.118.200190/-/DC1.

## References

[bib1] Anderson M. G. Smith R. S. Hawes N. L. Zabaleta A. Chang B., 2002 Mutations in genes encoding melanosomal proteins cause pigmentary glaucoma in DBA/2J mice. Nat. Genet. 30(1): 81–85. 10.1038/ng794 11743578

[bib2] Aptel F. Weinreb R. N. Chiquet C. Mansouri K., 2016 24-h monitoring devices and nyctohemeral rhythms of intraocular pressure. Prog. Retin. Eye Res. 55: 108–148. 10.1016/j.preteyeres.2016.07.002 27477112

[bib3] Bradley J. M. Vranka J. Colvis C. M. Conger D. M. Alexander J. P., 1998 Effect of matrix metalloproteinases activity on outflow in perfused human organ culture. Invest. Ophthalmol. Vis. Sci. 39(13): 2649–2658. 9856774

[bib4] Brubaker R. F., 1975 The effect of intraocular pressure on conventional outflow resistance in the enucleated human eye. Invest. Ophthalmol. 14(4): 286–292. 1123284

[bib5] Brubaker R. F., 1991 Flow of Aqueous-Humor in Humans - the Friedenwald Lecture. Invest. Ophthalmol. Vis. Sci. 32(13): 3145–3166. 1748546

[bib6] Burdon K. P. Macgregor S. Hewitt A. W. Sharma S. Chidlow G., 2011 Genome-wide association study identifies susceptibility loci for open angle glaucoma at TMCO1 and CDKN2B–AS1. Nat. Genet. 43(6): 574–578. 10.1038/ng.824 21532571

[bib7] Carbonaro F. Andrew T. Mackey D. A. Young T. L. Spector T. D., 2009 Repeated measures of intraocular pressure result in higher heritability and greater power in genetic linkage studies. Invest. Ophthalmol. Vis. Sci. 50(11): 5115–5119. 10.1167/iovs.09-3577 19420339 PMC4145813

[bib8] Carlborg O. De Koning D. J. Manly K. F. Chesler E. Williams R. W., 2005 Methodological aspects of the genetic dissection of gene expression. Bioinformatics 21(10): 2383–2393. 10.1093/bioinformatics/bti241 15613385

[bib9] Carnes M. U. Allingham R. R. Ashley-Koch A. Hauser M. A., 2018 Transcriptome analysis of adult and fetal trabecular meshwork, cornea, and ciliary body tissues by RNA sequencing. Exp. Eye Res. 167: 91–99. 10.1016/j.exer.2016.11.021 27914989

[bib10] Chang T. C. Congdon N. G. Wojciechowski R. Munoz B. Gilbert D., 2005 Determinants and heritability of intraocular pressure and cup-to-disc ratio in a defined older population. Ophthalmology 112(7): 1186–1191. 10.1016/j.ophtha.2005.03.006 15939473 PMC3124001

[bib11] Chen F. Klein A. P. Klein B. E. Lee K. E. Truitt B., 2014 Exome array analysis identifies CAV1/CAV2 as a susceptibility locus for intraocular pressure. Invest. Ophthalmol. Vis. Sci. 56(1): 544–551. 10.1167/iovs.14-15204 25525164 PMC4303040

[bib12] Chesler E. J. Lu L. Shou S. Qu Y. Gu J., 2005 Complex trait analysis of gene expression uncovers polygenic and pleiotropic networks that modulate nervous system function. Nat. Genet. 37(3): 233–242. 10.1038/ng1518 15711545

[bib13] Chintalapudi S. R. Maria D. Di Wang X. Bailey J. N. C. Hysi P. G. NEIGHBORHOOD consortium; International Glaucoma Genetics consortium, 2017 Systems genetics identifies a role for Cacna2d1 regulation in elevated intraocular pressure and glaucoma susceptibility. Nat. Commun. 8(1): 1755 10.1038/s41467-017-00837-5 29176626 PMC5701146

[bib14] Choquet H. Thai K. K. Yin J. Hoffmann T. J. Kvale M. N., 2017 A large multi-ethnic genome-wide association study identifies novel genetic loci for intraocular pressure. Nat. Commun. 8(1): 2108 10.1038/s41467-017-01913-6 29235454 PMC5727399

[bib15] Churchill G. A. Doerge R. W., 1994 Empirical threshold values for quantitative trait mapping. Genetics 138(3): 963–971. 7851788 10.1093/genetics/138.3.963 PMC1206241

[bib16] Cohen L. P. Pasquale L. R., 2014 Clinical characteristics and current treatment of glaucoma. Cold Spring Harb. Perspect. Med. 4(6): a017236 10.1101/cshperspect.a017236 24890835 PMC4031956

[bib17] Cone F. E. Gelman S. E. Son J. L. Pease M. E. Quigley H. A., 2010 Differential susceptibility to experimental glaucoma among 3 mouse strains using bead and viscoelastic injection. Exp. Eye Res. 91(3): 415–424. 10.1016/j.exer.2010.06.018 20599961 PMC2954410

[bib18] Ding C. Wang P. Tian N., 2011 Effect of general anesthetics on IOP in elevated IOP mouse model. Exp. Eye Res. 92(6): 512–520. 10.1016/j.exer.2011.03.016 21457709 PMC3116023

[bib19] Ethier C. R. Kamm R. D. Palaszewski B. A. Johnson M. C. Richardson T. M., 1986 Calculations of flow resistance in the juxtacanalicular meshwork. Invest. Ophthalmol. Vis. Sci. 27(12): 1741–1750. 3793404

[bib20] European Glaucoma Prevention Study, G., S. Miglior, N. Pfeiffer, V. Torri, T. Zeyen *et al*, 2007 Predictive factors for open-angle glaucoma among patients with ocular hypertension in the European Glaucoma Prevention Study. *Ophthalmology* 114 (1): 3–9. 10.1016/j.ophtha.2006.05.075 17070596

[bib21] Freeman N. E. Templeton J. P. Orr W. E. Lu L. Williams R. W., 2011 Genetic networks in the mouse retina: growth associated protein 43 and phosphatase tensin homolog network. Mol. Vis. 17: 1355–1372. 21655357 PMC3108897

[bib22] Gedde S. J. Panarelli J. F. Banitt M. R. Lee R. K., 2013 Evidenced-based comparison of aqueous shunts. Curr. Opin. Ophthalmol. 24(2): 87–95. 10.1097/ICU.0b013e32835cf0f5 23287104

[bib23] Geisert E. E. Lu L. Freeman-Anderson N. E. Templeton J. P. Nassr M., 2009 Gene expression in the mouse eye: an online resource for genetics using 103 strains of mice. Mol. Vis. 15: 1730–1763. 19727342 PMC2736153

[bib24] Goel M. Picciani R. G. Lee R. K. Bhattacharya S. K., 2010 Aqueous humor dynamics: a review. Open Ophthalmol. J. 4(1): 52–59. 10.2174/1874364101004010052 21293732 PMC3032230

[bib25] Gordon M. O. Beiser J. A. Brandt J. D. Heuer D. K. Higginbotham E. J., 2002 The Ocular Hypertension Treatment Study: baseline factors that predict the onset of primary open-angle glaucoma. *Arch Ophthalmol* 120 (6): 714–720; discussion 829–730 10.1001/archopht.120.6.714

[bib26] Herndon L. W. Weizer J. S. Stinnett S. S., 2004 Central corneal thickness as a risk factor for advanced glaucoma damage. Arch. Ophthalmol. 122(1): 17–21. 10.1001/archopht.122.1.17 14718289

[bib27] Hollows F. C. Graham P. A., 1966 Intra-ocular pressure, glaucoma, and glaucoma suspects in a defined population. Br. J. Ophthalmol. 50(10): 570–586. 10.1136/bjo.50.10.570 5954089 PMC506274

[bib28] Joe M. K. Sohn S. Hur W. Moon Y. Choi Y. R., 2003 Accumulation of mutant myocilins in ER leads to ER stress and potential cytotoxicity in human trabecular meshwork cells. Biochem. Biophys. Res. Commun. 312(3): 592–600. 10.1016/j.bbrc.2003.10.162 14680806

[bib29] Johnson M., 2006 ‘What controls aqueous humour outflow resistance?’. Exp. Eye Res. 82(4): 545–557. 10.1016/j.exer.2005.10.011 16386733 PMC2892751

[bib30] Kasetti R. B. Phan T. N. Millar J. C. Zode G. S., 2016 Expression of Mutant Myocilin Induces Abnormal Intracellular Accumulation of Selected Extracellular Matrix Proteins in the Trabecular Meshwork. Invest. Ophthalmol. Vis. Sci. 57(14): 6058–6069. 10.1167/iovs.16-19610 27820874 PMC5102566

[bib31] King R. Lu L. Williams R. W. Geisert E. E., 2015 Transcriptome networks in the mouse retina: An exon level BXD RI database. Mol. Vis. 21: 1235–1251. 26604663 PMC4626778

[bib32] Klein B. E. Klein R. Lee K. E., 2004 Heritability of risk factors for primary open-angle glaucoma: the Beaver Dam Eye Study. Invest. Ophthalmol. Vis. Sci. 45(1): 59–62. 10.1167/iovs.03-0516 14691154

[bib33] Kumar P. Henikoff S. Ng P. C., 2009 Predicting the effects of coding non-synonymous variants on protein function using the SIFT algorithm. Nat. Protoc. 4(7): 1073–1081. 10.1038/nprot.2009.86 19561590

[bib34] Leske M. C. Heijl A. Hyman L. Bengtsson B. Dong L., 2007 Predictors of long-term progression in the early manifest glaucoma trial. Ophthalmology 114(11): 1965–1972. 10.1016/j.ophtha.2007.03.016 17628686

[bib35] Liton P. B. Luna C. Challa P. Epstein D. L. Gonzalez P., 2006 Genome-wide expression profile of human trabecular meshwork cultured cells, nonglaucomatous and primary open angle glaucoma tissue. Mol. Vis. 12: 774–790. 16862071 PMC3152462

[bib36] Medeiros F. A. Sample P. A. Zangwill L. M. Bowd C. Aihara M., 2003 Corneal thickness as a risk factor for visual field loss in patients with preperimetric glaucomatous optic neuropathy. Am. J. Ophthalmol. 136(5): 805–813. 10.1016/S0002-9394(03)00484-7 14597030

[bib37] Mozhui K. Ciobanu D. C. Schikorski T. Wang X. Lu L., 2008 Dissection of a QTL hotspot on mouse distal chromosome 1 that modulates neurobehavioral phenotypes and gene expression. PLoS Genet. 4(11): e1000260 10.1371/journal.pgen.1000260 19008955 PMC2577893

[bib38] Nag A. Venturini C. Small K. S. Young T. L. Viswanathan A. C. International Glaucoma Genetics Consortium, 2014 A genome-wide association study of intra-ocular pressure suggests a novel association in the gene FAM125B in the TwinsUK cohort. Hum. Mol. Genet. 23(12): 3343–3348. 10.1093/hmg/ddu050 24518671 PMC4030784

[bib39] Ojha P. Wiggs J. L. Pasquale L. R., 2013 The genetics of intraocular pressure. Semin. Ophthalmol. 28(5–6): 301–305. 10.3109/08820538.2013.825291 24138038

[bib40] Ozel A. B. Moroi S. E. Reed D. M. Nika M. Schmidt C. M., 2014 Genome-wide association study and meta-analysis of intraocular pressure. Hum. Genet. 133(1): 41–57. 10.1007/s00439-013-1349-5 24002674 PMC3982323

[bib41] Paylakhi S. H. Yazdani S. April C. Fan J. B. Moazzeni H., 2012 Non-housekeeping genes expressed in human trabecular meshwork cell cultures. Mol. Vis. 18: 241–254. 22312193 PMC3272053

[bib42] Peirce J. L. Lu L. Gu J. Silver L. M. Williams R. W., 2004 A new set of BXD recombinant inbred lines from advanced intercross populations in mice. BMC Genet. 5(1): 7 10.1186/1471-2156-5-7 15117419 PMC420238

[bib43] Qiu Y. Yang H. Lei B., 2014 Effects of three commonly used anesthetics on intraocular pressure in mouse. Curr. Eye Res. 39(4): 365–369. 10.3109/02713683.2013.845224 24215504

[bib44] Renard E. Palombi K. Gronfier C. Pepin J. L. Noel C., 2010 Twenty-four hour (Nyctohemeral) rhythm of intraocular pressure and ocular perfusion pressure in normal-tension glaucoma. Invest. Ophthalmol. Vis. Sci. 51(2): 882–889. 10.1167/iovs.09-3668 19684006

[bib45] Rosen G. D. La Porte N. T. Diechtiareff B. Pung C. J. Nissanov J., 2003 Informatics center for mouse genomics: the dissection of complex traits of the nervous system. Neuroinformatics 1(4): 327–342. 10.1385/NI:1:4:327 15043219

[bib46] Row S. Liu Y. Alimperti S. Agarwal S. K. Andreadis S. T., 2016 Cadherin-11 is a novel regulator of extracellular matrix synthesis and tissue mechanics. J. Cell Sci. 129(15): 2950–2961. 10.1242/jcs.183772 27311482 PMC5004872

[bib47] Samsel P. A. Kisiswa L. Erichsen J. T. Cross S. D. Morgan J. E., 2011 A novel method for the induction of experimental glaucoma using magnetic microspheres. Invest. Ophthalmol. Vis. Sci. 52(3): 1671–1675. 10.1167/iovs.09-3921 20926815

[bib48] Sappington R. M. Carlson B. J. Crish S. D. Calkins D. J., 2010 The microbead occlusion model: a paradigm for induced ocular hypertension in rats and mice. Invest. Ophthalmol. Vis. Sci. 51(1): 207–216. 10.1167/iovs.09-3947 19850836 PMC2869054

[bib49] Savinova O. V. Sugiyama F. Martin J. E. Tomarev S. I. Paigen B. J., 2001 Intraocular pressure in genetically distinct mice: an update and strain survey. BMC Genet. 2(1): 12 10.1186/1471-2156-2-12 11532192 PMC48141

[bib50] Sommer A. Tielsch J. M. Katz J. Quigley H. A. Gottsch J. D., 1991 Relationship between intraocular pressure and primary open angle glaucoma among white and black Americans. The Baltimore Eye Survey. Arch. Ophthalmol. 109(8): 1090–1095. 10.1001/archopht.1991.01080080050026 1867550

[bib51] Springelkamp H. Höhn R. Mishra A. Hysi P. G. Khor C. C., 2014 Meta-analysis of genome-wide association studies identifies novel loci that influence cupping and the glaucomatous process. Nat. Commun. 5: 4883 10.1038/ncomms5883 25241763 PMC4199103

[bib52] Springelkamp H. Iglesias A. I. Mishra A. Hohn R. Wojciechowski R., 2017 New insights into the genetics of primary open-angle glaucoma based on meta-analyses of intraocular pressure and optic disc characteristics. Hum. Mol. Genet. 26(2): 438–453. 10.1093/hmg/ddw399 28073927 PMC5968632

[bib53] Stone E. M. Fingert J. H. Alward W. L. Nguyen T. D. Polansky J. R., 1997 Identification of a gene that causes primary open angle glaucoma. Science 275(5300): 668–670. 10.1126/science.275.5300.668 9005853

[bib54] Struebing F. L. Geisert E. E., 2015 What Animal Models Can Tell Us About Glaucoma. Prog. Mol. Biol. Transl. Sci. 134: 365–380. 10.1016/bs.pmbts.2015.06.003 26310165

[bib55] Struebing F. L. King R. Li Y. Chrenek M. A. Lyuboslavsky P. N., 2018 a Transcriptional Changes in the Mouse Retina after Ocular Blast Injury: A Role for the Immune System. J. Neurotrauma 35(1): 118–129. 10.1089/neu.2017.5104 28599600 PMC5757081

[bib56] Struebing F. L. King R. Li Y. Cooke Bailey J. N. Wiggs J. L. NEIGHBORHOOD consortium, 2018 b Genomic loci modulating retinal ganglion cell death following elevated IOP in the mouse. Exp. Eye Res. 169: 61–67. 10.1016/j.exer.2017.12.013 29421330 PMC5939594

[bib57] Sun N. Shibata B. Hess J. F. FitzGerald P. G., 2015 An alternative means of retaining ocular structure and improving immunoreactivity for light microscopy studies. Mol. Vis. 21: 428–442. 25991907 PMC4403009

[bib58] Takahashi H. Noda S. Imamura Y. Nagasawa A. Kubota R., 1998 Mouse myocilin (Myoc) gene expression in ocular tissues. Biochem. Biophys. Res. Commun. 248(1): 104–109. 10.1006/bbrc.1998.8917 9675094

[bib59] Templeton J. P. Freeman N. E. Nickerson J. M. Jablonski M. M. Rex T. S., 2013 Innate immune network in the retina activated by optic nerve crush. Invest. Ophthalmol. Vis. Sci. 54(4): 2599–2606. 10.1167/iovs.12-11175 23493296 PMC3629889

[bib60] van Koolwijk L. M. Despriet D. D. van Duijn C. M. Pardo Cortes L. M. Vingerling J. R., 2007 Genetic contributions to glaucoma: heritability of intraocular pressure, retinal nerve fiber layer thickness, and optic disc morphology. Invest. Ophthalmol. Vis. Sci. 48(8): 3669–3676. 10.1167/iovs.06-1519 17652737

[bib61] van Koolwijk L. M. Ramdas W. D. Ikram M. K. Jansonius N. M. Pasutto F., 2012 Common genetic determinants of intraocular pressure and primary open-angle glaucoma. PLoS Genet. 8(5): e1002611 10.1371/journal.pgen.1002611 22570627 PMC3342933

[bib62] Vranka J. A. Kelley M. J. Acott T. S. Keller K. E., 2015 Extracellular matrix in the trabecular meshwork: intraocular pressure regulation and dysregulation in glaucoma. Exp. Eye Res. 133: 112–125. 10.1016/j.exer.2014.07.014 25819459 PMC4379427

[bib63] Wang W. H. Millar J. C. Pang I. H. Wax M. B. Clark A. F., 2005 Noninvasive measurement of rodent intraocular pressure with a rebound tonometer. Invest. Ophthalmol. Vis. Sci. 46(12): 4617–4621. 10.1167/iovs.05-0781 16303957

[bib64] Wiggs J. L., 2007 Genetic etiologies of glaucoma. Arch. Ophthalmol. 125(1): 30–37. 10.1001/archopht.125.1.30 17210849

[bib65] Zhou E. H. Krishnan R. Stamer W. D. Perkumas K. M. Rajendran K., 2012 Mechanical responsiveness of the endothelial cell of Schlemm’s canal: scope, variability and its potential role in controlling aqueous humour outflow. J. R. Soc. Interface 9(71): 1144–1155. 10.1098/rsif.2011.0733 22171066 PMC3350739

